# Dynamical system of the growth of COVID-19 with controller

**DOI:** 10.1186/s13662-020-03168-w

**Published:** 2021-01-07

**Authors:** Rabha W. Ibrahim, Dania Altulea, Rafida M. Elobaid

**Affiliations:** 1 Informetrics Research Group, Ton Duc Thang University, Ho Chi Minh City, Vietnam; 2 Faculty of Mathematics & Statistics, Ton Duc Thang University, Ho Chi Minh City, Vietnam; 3 Faculty of Science, University of Groningen, Groningen, The Netherlands; 4 Department of General Sciences, Prince Sultan University, Riyadh, Saudi Arabia

**Keywords:** Conformable calculus, Fractional calculus, Dynamic system, COVID-19

## Abstract

Recently, various studied were presented to describe the population dynamic of covid-19. In this effort, we aim to introduce a different vitalization of the growth by using a controller term. Our method is based on the concept of conformable calculus, which involves this term. We investigate a system of coupled differential equations, which contains the dynamics of the diffusion among infected and asymptomatic characters. Strong control is considered due to the social separation. The result is consequently associated with a macroscopic law for the population. This dynamic system is useful to recognize the behavior of the growth rate of the infection and to confirm if its control is correctly functioning. A unique solution is studied under self-mapping properties. The periodicity of the solution is examined by using integral control and the optimal control is discussed in the sequel.

## Introduction

COVID-19 has been spreading speedily into many countries in the world; the World Health Organization (WHO) classified it as a pandemic. The first WHO report of dyed-in-the-wool cases of COVID-19 was issued on January 21, 2020 with 282 confirmed cases, which is outstanding with the most recent report on March 18, 2020, which mentions 191,127 confirmed cases (see [1, 2]). Numerous growth designs have been very newly employed to describe the time evolution of the COVID-19 infection [3]. Mainly, by utilizing the system $d\chi (t) /dt=\chi (t) $, where *χ* represents the number of infected people, the rampant phase, the increasing number of asymptomatic infected persons, is described. Nowadays, there are different numerical studies and analytic investigations in COVID-19 have been presented (see [4–7]).

The present work deals with the generalized dynamic system of the growth laws by applying the concept of conformable calculus. This concept involves an important term which is the controller to organize and expect the graph of the growth. The existence and uniqueness of the solution are studied in view of the fixed point theory of self-mappings. Other properties are investigated such as the integrated and optimal controller.

## Conformable dynamic system (CDS)

In this section, we construct the dynamic system of a couple equations. Before that, we need the following preliminaries about the conformable calculus [8].

### Conformable calculus (CC)

In this part, we introduce the definition of the conformable calculus.

#### Definition 1

(Conformable differential operator)

A differential operator ${\mathcal{D}}^{\nu }, \nu \in [0,1]$, is conformable if and only if ${\mathcal{D}}^{0}$ is the identity operator and ${\mathcal{D}}^{1}$ is the ordinary differential operator. Particularly, the operator is conformable if and only if a differential function $\chi (t)$ satisfies

$$ {\mathcal{D}}^{0}\chi (t)=\chi (t)\quad \text{and} \quad{\mathcal{D}}^{1}\chi (t)=\frac{d}{dt}\chi (t)=\chi '(t). $$

In the theory of control systems, a proportional-differential controller for controlling resultant *υ* at time *t* with two tuning criteria has the setting

1 $$ \upsilon (t)={\sigma }_{p}\Sigma (t)+{\sigma }_{d}\frac{d}{dt}\Sigma (t), $$

where ${\sigma }_{p}$ is the proportional gain, ${\sigma }_{d}$ is the derivative gain, and Σ is the error between the formal variable and the actual variable. Based on (1), Anderson and Ulness [9] developed the common idea of CC.

#### Definition 2

(A special class of conformable calculus)

For two continuous functions $\sigma _{0}, \sigma _{1}: [0,1]\times \mathbb{R}\rightarrow (0, \infty )$, we obtain

2 $$ {\mathcal{D}}^{\nu }\chi (t) =\sigma _{1}(\nu,t) \chi (t)+\sigma _{0} ( \nu,t) \chi '(t), $$

where

$$ \lim_{\nu \rightarrow 0}\sigma _{1}(\nu,t)=1,\qquad \lim _{\nu \rightarrow 1}\sigma _{1}(\nu,t)=0,\quad \sigma _{1}( \nu,t)\neq 0, \forall t, {\nu } {\in } (0,1), $$

and

$$ \lim_{\nu \rightarrow 0}\sigma _{0}(\beta,t)=0,\qquad \lim _{\nu \rightarrow 1}\sigma _{0}(\nu,t)=1,\quad \sigma _{0}( \nu,t)\neq 0, \forall t, {\nu } {\in } (0,1). $$

#### Definition 3

The integral operator corresponding to $\mathcal{D}^{\nu }$ has the following expression:

3 $$ \int \mathcal{D}^{\nu }\chi (t) \,d_{\nu }t=\chi (t)+k e_{0}(t,t_{0}), $$

where $k \in \mathbb{R}$, $d_{\nu }t=\frac{dt}{\sigma _{0}(t)}, \nu \neq 0$, and

4 $$ e_{0}(t,\kappa )= \exp \biggl(- \int _{\kappa }^{t} \frac{\sigma _{1}(\nu,\varsigma )}{\sigma _{0}(\nu,\varsigma ))}\,d \varsigma \biggr). $$

Also, the definite integral of the derivative of *χ* over the interval $[a, b] $ is given by

$$ \int _{a}^{t}\bigl[ \mathcal{D}^{\nu }\chi ( \varsigma )\bigr] e_{0}(t,\varsigma ) \,d_{\nu }\varsigma =\chi (t)-\chi (a)e_{0}(t,a). $$

In our investigation, we need one of the following formulas of $\sigma _{1}$ and $\sigma _{0}$:

5 $$\begin{aligned} &\sigma _{1}(\nu,t)= (1-\nu ) t^{\nu },\qquad \sigma _{0}(\nu,t)= \nu t^{1- \nu },\quad t \in (0, \infty ), \end{aligned}$$

6 $$\begin{aligned} &\sigma _{1}(\nu,t)= (1-\nu ) |t|^{\nu },\qquad \sigma _{0}( \nu,t)= \nu |t|^{1-\nu }, \end{aligned}$$

7 $$\begin{aligned} &\sigma _{1}(\nu,t)= \cos \biggl(\frac{\nu \pi }{2}\biggr) t^{\nu },\qquad \sigma _{0}( \nu,t)=\sin \biggl(\frac{\nu \pi }{2} \biggr) t^{1-\nu }, \quad t \in (0,\infty ), \end{aligned}$$

8 $$\begin{aligned} &\sigma _{1}(\nu,t)= \cos \biggl(\frac{\nu \pi }{2}\biggr) |t|^{\nu }, \qquad \sigma _{0}( \nu,t)=\sin \biggl(\frac{\nu \pi }{2} \biggr)| t|^{1-\nu }, \quad t \in \mathbb{R} \backslash \{0\}, \end{aligned}$$

or for $\psi _{0},\psi _{1} \in (0,\infty )$

9 $$ \sigma _{0}(\nu,t)= \nu \psi _{0}^{1-\nu },\qquad \sigma _{1}(\nu,t)= (1-\nu )\psi _{1}^{\nu }. $$

Lastly, the conformable inner product between two continuous functions *χ* and *υ* has the formula

$$ \langle\chi,\upsilon \rangle = \int _{a}^{b}\chi (t)\upsilon (t) e_{0}(b,t)\,d_{\nu }t. $$

### Construction of CDS

**Figure 1:**
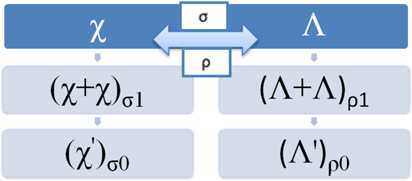
The dynamic process of system (10) with the connections

In the structure of CDS, we require $\Xi (t) $, the growing overall number of infected individuals, which is the sum of the number of the growing identified infected persons $\chi (t)$ and of the asymptomatic transmission ones $\Lambda (t)$: $\Xi (t) = \chi (t) + \Lambda (t)$. On the information of $\chi (t)$ the number of passing on the decease, the process of curing people is involved, because they have been formerly diseased. The rate of each function is given by the conformable connections $\sigma _{0}$ and $\sigma _{1}$ for the conformable changing in $\chi (t)$ and $\rho _{0}$ and $\rho _{1}$ for the conformable changing in $\Lambda (t)$ satisfying Definition 2. Based on the above discussion, we have the following CDS:

10 $$ \begin{aligned} & {\mathcal{D}}^{\nu }\chi (t) = \sigma _{1}(\nu,t) \chi (t)+ \sigma _{0} (\nu,t) \chi '(t)+ \sigma (t) \Lambda (t), \\ & {\mathcal{D}}^{\mu }\Lambda (t) =\rho _{1}(\mu,t) \Lambda (t)+\rho _{0} (\mu,t) \Lambda '(t)+ \rho (t) \chi (t), \end{aligned} $$

where *σ* and *ρ* are the connection rate functions of Λ in ${\mathcal{D}}^{\nu }\chi (t)$ and *χ* in ${\mathcal{D}}^{\mu }\Lambda (t)$, respectively. They describe the damping properties in line for the control energy. The dynamic process of (10) can be recognized in Fig. 1.

There are two directions to study CDS in view of the mathematical analysis: parametric and bifurcation analysis. Parametric analysis considers the measurable adjustments of path dynamics in reaction to perturbations of path parameters. This analysis is typically employed for identifying critical path methods. Therefore, there are diverse procedures to seek the performance of the system, such as critical point theory, and fixed point theory. Centering on the second option, we employ the current fixed point theorem of a self-mapping. Meanwhile, bifurcation analysis is applied to control how qualitative properties of a path rest on its parameters. Explicitly, it studies the steady-state results of a structure and their stability.

## Results

In this section, we proceed to discuss the existence and uniqueness solution of system (10). Moreover, we investigate the controller solution from different points of view.

### Unique solution

By assuming $X(t,\chi ):=\chi '(t)$ and $A(t,\chi ):=\Lambda '(t)$, we obtain the following system:

11 $$ \begin{aligned} & {\mathcal{D}}^{\nu }\chi (t) = \sigma _{1}(\nu,t) \chi (t)+ \sigma _{0} (\nu,t) X(t,\chi )+ \sigma (t) \Lambda (t), \\ & {\mathcal{D}}^{\mu }\Lambda (t) =\rho _{1}(\mu,t) \Lambda (t)+\rho _{0} (\mu,t) A(t,\Lambda )+ \rho (t) \chi (t), \end{aligned} $$

satisfying the following assumptions:

1. Assume that $X: [0,T]\times \mathbb{R} \rightarrow \mathbb{R}$ is a non-decreasing continuously differentiated function with $X (0,0) =0$ and non-vanishing in a compact interval $(0, T] $. Furthermore, there is a positive constant *ℓ* such that
  $$ \bigl|X(t,\chi _{1})-X(t,\chi _{2})\bigr| \leq \ell |\chi _{1}-\chi _{2}|. $$
2. Assume that $A: [0,T]\times \mathbb{R} \rightarrow \mathbb{R}$ is a non-decreasing continuously differentiated function with $A (0,0) =0$ and non-vanishing in a compact interval $(0, T]$; in addition, assume that there exists a positive constant *L* such that
  $$ \bigl|A(t,\Lambda _{1})-A(t,\Lambda _{2})\bigr| \leq L \bigl|\Lambda _{1}- \Lambda _{2}\bigr|. $$

We aim to establish the existence and the uniqueness solution of system (11) using self-mapping fixed point theorem [10].

#### Lemma 3.1

*Let* $(M,\vartriangle )$ *be a complete metric space and* $\mathcal{W}\colon M\to M$ *a self*-*mapping leading to the relation*

12 $$\begin{aligned} \flat \bigl(\vartriangle \bigl(\mathcal{W}(x), \mathcal{W}(y)\bigr) \bigr) \leq \flat \bigl(\vartriangle (x,y)\bigr)-\wp \bigl( \vartriangle (x,y) \bigr) \end{aligned}$$

*for all* $x,y\in M$, *where* $\flat,\wp \colon [0,\infty )\to [0,\infty )$ *are both continuous and non*-*decreasing functions with* $\flat (0)=\wp (0)=0$. *Then* $\mathcal{W}$ *admits a unique fixed point*.

Let $M=\mathbb{R}$ and define an operator $\mathcal{P}: \mathbb{R} \times \mathbb{R} \rightarrow \mathbb{R} \times \mathbb{R}$ as follows:

13 $$\begin{aligned} \bigl(\mathcal{P}( \chi,\Lambda ) \bigr) (t)={}& \bigl(\mathcal{P}_{1}( \chi, \Lambda ), \mathcal{P}_{2}( \chi,\Lambda ) \bigr) (t) \\ \begin{aligned} ={}& ( \int \bigl( \sigma _{1}(\nu,t) \chi (t)+\sigma _{0} ( \nu,t) X\bigl(t, \chi (t)\bigr)+\sigma (t) \Lambda (t) \bigr) \,d_{\nu }t+c_{1}, \\ &{}\times \int \bigl( \rho _{1}(\mu,t) \Lambda (t)+\rho _{0} ( \mu,t) A\bigl(t, \Lambda (t)+ \rho (t) \chi (t) \bigr) \,d_{\nu }t+c_{2} \bigr). \end{aligned} \end{aligned}$$

Since $(\chi,\Lambda ) \in \mathbb{R}\times \mathbb{R,}$ $\mathcal{P}$ is a self-mapping.

#### Lemma 3.2

*Consider the functions* $\mathfrak{G}_{1,2}: \mathbb{R}^{3} \rightarrow \mathbb{R}^{+}$ *by*

$$ \mathfrak{G}_{1}(\chi _{1},\chi _{2},\chi _{3})= \max \bigl\{ | \chi _{\imath }-\chi _{\jmath }|:\imath, \jmath =1,2,3, \imath \neq \jmath\bigr\} $$

*and*

$$ \mathfrak{G}_{2}(\Lambda _{1},\Lambda _{2}, \Lambda _{3})= \max \bigl\{ | \Lambda _{\imath }-\Lambda _{\jmath }|: \imath,\jmath =1,2,3, \imath \neq \jmath\bigr\} . $$

*Then the function* $\mathfrak{G}:=(\mathfrak{G}_{1},\mathfrak{G}_{2}) \in \mathbb{R} \times \mathbb{R}$ *forms a metric*.

#### Proof

Clearly, $\mathfrak{G}_{1}(\chi _{1},\chi _{2},\chi _{3})=0$ for $\chi _{1}=\chi _{2}=\chi _{3}$; moreover, we get the following calculation:

14 $$ \begin{aligned} &\mathfrak{G}_{1}(\chi _{1},\chi _{1},\chi _{i})+ \mathfrak{G}_{1}( \chi _{2},\chi _{2},\chi _{j})+ \mathfrak{G}_{1}( \chi _{3},\chi _{3},\chi _{k}) \\ &\quad= \max_{i=2,3}\bigl\{ | \chi _{1}-\chi _{i}|\bigr\} + \max_{j=1,3}\bigl\{ | \chi _{2}- \chi _{j}|\bigr\} + \max_{k=1,2}\bigl\{ | \chi _{3}-\chi _{k}|\bigr\} \\ &\quad= \max \bigl\{ | \chi _{1}-\chi _{2}|,| \chi _{1}-\chi _{3}\bigr\} + \max \bigl\{ | \chi _{2}-\chi _{1}|,| \chi _{2}-\chi _{3}\bigr\} \\ &\qquad{} + \max \bigl\{ | \chi _{3}-\chi _{1}|,| \chi _{3}- \chi _{2}|\bigr\} \\ &\quad= 2 \max \bigl\{ | \chi _{1}-\chi _{2}|, | \chi _{2}- \chi _{3}|,| \chi _{3}- \chi _{1}|\bigr\} \\ &\quad> \max \bigl\{ | \chi _{1}-\chi _{2}|, | \chi _{2}- \chi _{3}|,| \chi _{3}- \chi _{1}|\bigr\} \\ &\quad= \max \bigl\{ | \chi _{\imath }-\chi _{\jmath }|: \imath,\jmath =1,2,3, \imath \neq \jmath\bigr\} \\ &\quad=\mathfrak{G}_{1}(\chi _{1},\chi _{2},\chi _{3}). \end{aligned} $$

Hence, the function $\mathfrak{G}_{1}(\chi _{1},\chi _{2},\chi _{3})$ is a metric on the set $\mathbb{R}$. Similarly for $\mathfrak{G}_{2} \in \mathbb{R}$. We conclude that $\mathfrak{G} \in \mathbb{R}\times \mathbb{R}$, which indicates a metric. □

This metric indicates the maximum measurement between the three cases of the growth of covid-19 (Fig. 1, the first column for *χ* and the second column for Λ). Note that this metric can extend to include other cases in the dynamical systems.

#### Theorem 3.3

*Consider the dynamic system* (11) *satisfying the assumptions* (A1) *and* (A2). *If the positive constants* *ℓ* *and* *L* *obey*

$$ \ell < \frac{1-(1-\nu )T^{\nu }}{\nu T^{1-\nu }} \quad\textit{and}\quad L< \frac{1-(1-\mu ) T^{\mu }}{\mu T^{1-\mu }}, T< \infty, $$

*respectively*, *then* $\mathcal{P}$ *has a unique fixed point in the ball* $B_{r}:=(B_{r1},B_{r2})$, *where* $r_{1}\leq 1$ *and* $r_{2}\leq 1$.

#### Proof

Let the functions $\sigma _{0}$ and $\sigma _{1}$ be

$$ \sigma _{1}(\nu,t)= (1-\nu ) t^{\nu },\qquad \sigma _{0}( \nu,t)= \nu t^{1- \nu },\quad t \in (0, T), T< \infty $$

and

$$ \rho _{1}(\mu,t)= (1-\mu ) t^{\mu }, \qquad \rho _{0}( \mu,t)= \mu t^{1- \mu },\quad t \in (0, T), T< \infty. $$

Note that a similar proof can be presented for other formulas. Then, by the assumption on *ℓ*, and the definition of the metric in Lemma 3.2, we have

$$ \begin{aligned} & \mathfrak{G}_{1}\bigl(\mathcal{P}_{1} \chi _{1}(t), \mathcal{P}_{1} \chi _{2}(t), \mathcal{P}_{1}\chi _{3}(t)\bigr) \\ &\quad= \max \bigl\{ \bigl| \mathcal{P}_{1}\chi _{\imath }(t)- \mathcal{P}_{1}\chi _{\jmath }(t)\bigr|: \imath,\jmath =1,2,3, \imath \neq \jmath \bigr\} \\ &\quad \leq \max \biggl\{ \bigl|\sigma _{1}(\nu,t) \chi _{\imath }(t)+\sigma _{0} (\nu,t) X(t,\chi _{\imath }) \\ &\qquad{} - \bigl( \sigma _{1}(\nu,t) \chi _{\jmath }(t)+\sigma _{0} ( \nu,t) X\bigl(t,\chi _{\jmath }(t)\bigr) \bigr) \bigl| \frac{T^{\nu }}{\nu ^{2}}: \imath,\jmath =1,2,3, \imath \neq \jmath \biggr\} \\ &\quad\leq \max \biggl\{ \bigl(\sigma _{1}(\nu,t)| \chi _{\imath }- \chi _{\jmath }| + \sigma _{0}(\nu,t)\ell | \chi _{\imath }- \chi _{\jmath }| \bigr) \frac{T^{\nu }}{\nu ^{2}}: \imath,\jmath =1,2,3, \imath \neq \jmath \biggr\} \\ &\quad\leq \max \biggl\{ \bigl((1-\nu ) T^{\nu }| \chi _{\imath }-\chi _{\jmath }| + \nu T^{1-\nu }\ell | \chi _{\imath }-\chi _{\jmath }| \bigr) \frac{T^{\nu }}{\nu ^{2}}: \imath,\jmath =1,2,3, \imath \neq \jmath \biggr\} \\ &\quad= \max \biggl\{ \bigl[(1-\nu ) T^{\nu }+ \nu T^{1-\nu } \ell \bigr] \frac{T^{\nu }}{\nu ^{2}} | \chi _{\imath }-\chi _{\jmath }|: \imath, \jmath =1,2,3, \imath \neq \jmath \biggr\} \\ &\quad= \bigl[(1-\nu ) T^{\nu }+ \nu T^{1-\nu } \ell \bigr] \frac{T^{\nu }}{\nu ^{2}} \bigl( \max \bigl\{ | \chi _{\imath }-\chi _{\jmath }|: \imath,\jmath =1,2,3, \imath \neq \jmath\bigr\} \bigr) \\ &\quad:=r_{1} \mathfrak{G}_{1}(\chi _{1},\chi _{2},\chi _{3}). \end{aligned} $$

By the assumption of the theorem on *ℓ*, we have

$$ \begin{aligned} & \bigl[(1-\nu ) T^{\nu }+ \nu T^{1-\nu } \ell \bigr] \frac{T^{\nu }}{\nu ^{2}}< 1 \\ &\quad \Rightarrow \quad\bigl[(1-\nu ) T^{\nu }+\nu T^{1-\nu } \ell \bigr]< \frac{\nu ^{2}}{T^{\nu }} \\ &\quad < 1, \end{aligned} $$

which leads to the boundedness of the operator $\mathcal{P}_{1}$ in the unit ball $B_{r1} $ of radius $0 < r_{1}<1$. Similarly for $\mathcal{P}_{2}$, which is bounded in the ball $B_{r2}, 0< r_{2}<1 $. Combining the above conclusions, we find that the operator $\mathcal{P}=(\mathcal{P}_{1},\mathcal{P}_{2})$ is bounded in $B_{r}= (B_{r1}, B_{r2}) $.

We proceed to discover more properties of the operator $\mathcal{P}_{1}$. For $t,\tau \in (0,T)$ with $t>\tau $ and $\chi (t)>\chi (\tau )$ (increasing function), we have

$$\begin{aligned} & \mathfrak{G}_{1} (\mathcal{P}_{1} \chi _{1}(t), \mathcal{P}_{1}\chi _{2}(t), \mathcal{P}_{1}\chi _{3}(t) -\bigl(\mathcal{P}_{1} \chi _{1}(\tau ),\mathcal{P}_{1}\chi _{2}(\tau ), \mathcal{P}_{1}\chi _{3}( \tau ) \bigr) \\ &\quad= \mathfrak{G}_{1} \bigl(\mathcal{P}_{1} \bigl(\chi _{1}(t)-\chi _{1}( \tau ) \bigr),\mathcal{P}_{1} \bigl(\chi _{2}(t)-\chi _{2}(\tau ) \bigr), \mathcal{P}_{1} \bigl(\chi _{3}(t)-\chi _{3}(\tau ) \bigr) \bigr) \\ &\quad= \mathfrak{G}_{1} \bigl(\mathcal{P}_{1}\chi _{1}(t-\tau ),\mathcal{P}_{1} \chi _{2}(t-\tau ), \mathcal{P}_{1}\chi _{3}(t-\tau ) \bigr) \\ &\quad\leq \mathfrak{G}_{1} \bigl(\mathcal{P}_{1}\chi _{1}(t),\mathcal{P}_{1} \chi _{2}(t), \mathcal{P}_{1}\chi _{3}(t) \bigr) \\ &\quad\leq r_{1} \mathfrak{G}_{1}(\chi _{1},\chi _{2},\chi _{3}). \end{aligned}$$

Thus, $\mathcal{P}_{1}$ is equicontinuous on $B_{r1}$. Similarly for $\mathcal{P}_{2}$; thus, the integral operator $\mathcal{P}$ is equicontinuous on $B_{r}$. Next, we check the continuity of the integral operator $\mathcal{P}\in B_{r}$.

Now, by putting $\chi _{l}(t)-\eta _{l}(t)=\xi _{l}(t)$, $l=1,2,3$, we obtain

$$ \begin{aligned} & \mathfrak{G}_{1} \bigl( \mathcal{P}_{1} \bigl(\chi _{1}(t)- \eta _{1}(t) \bigr),\mathcal{P}_{1} \bigl(\chi _{2}(t)-\eta _{2}(t) \bigr), \mathcal{P}_{1} \bigl(\chi _{3}(t)-\eta _{3}(t) \bigr) \bigr) \\ &\quad= \mathfrak{G}_{1} \bigl( \mathcal{P}_{1} \bigl(\xi _{1}(t) \bigr), \mathcal{P}_{1} \bigl(\xi _{2}(t) \bigr),\mathcal{P}_{1} \bigl(\xi _{3}(t) \bigr) \bigr) \\ & \quad\leq \max \biggl\{ \bigl|\sigma _{1}(\nu,t) \xi _{\imath }(t)+\sigma _{0} (\nu,t) X(\xi _{\imath }\bigl( \chi _{\imath }(t)\bigr) \\ &\qquad{} - (\sigma _{1}(\nu,t)\xi _{\jmath }(t)+\sigma _{0} ( \nu,t) X\bigl(\xi _{\jmath }\bigl( \xi _{\jmath }(t)\bigr) \bigr) \bigr| \frac{T^{\nu }}{\nu ^{2}}: \imath,\jmath =1,2,3, \imath \neq \jmath \biggr\} \\ &\quad\leq \max \biggl\{ \sigma _{1}(\nu,t)| \xi _{\imath }-\xi _{\jmath }| \frac{T^{\nu }}{\nu ^{2}}+\sigma _{0} \ell | \xi _{\imath }-\xi _{\jmath }| \frac{T^{\nu }}{\nu ^{2}}: \imath,\jmath =1,2,3, \imath \neq \jmath \biggr\} \\ &\quad\leq \max \biggl\{ (1-\nu )T^{\nu } | \xi _{\imath }-\xi _{\jmath }| \frac{T^{\nu }}{\nu ^{2}}+\nu T^{1-\nu } \ell | \xi _{\imath }-\xi _{\jmath }| \frac{T^{\nu }}{\nu ^{2}}: \imath,\jmath =1,2,3, \imath \neq \jmath \biggr\} \\ &\quad= \max \biggl\{ \bigl[(1-\nu ) T ^{\nu }+\nu T^{1-\nu }(\nu,t)\ell \bigr] \frac{T^{\nu }}{\nu ^{2}} | \xi _{\imath }-\xi _{\jmath }|: \imath,\jmath =1,2,3, \imath \neq \jmath \biggr\} \\ &\quad= r_{1} \mathfrak{G}_{1} (\xi _{1},\xi _{2},\xi _{3}) \leq r_{1} \mathfrak{G}_{1}( \chi _{1},\chi _{2},\chi _{3}). \end{aligned} $$

Therefore, the operator $\mathcal{P}_{1}$ is continuous in $B_{r1}$. Similarly, for $\mathcal{P}_{2}$, which leads to $\mathcal{P}$ having a fixed point $\mathcal{P}(\chi,\Lambda )=(\chi,\Lambda )$ corresponding to the solution of the dynamic system (11).

Next, we aim to satisfy inequality (12). Suppose that there are two continuous and non-decreasing functions $\flat,\wp \colon [0,\infty )\to [0,\infty )$ having the properties: $\flat (t),\wp (t)>0$ for $t>0$ and $\flat (0)=\wp (0)=0$. Now, suppose that

$$ \flat (\epsilon )=\epsilon /r_{1},\qquad \wp (\epsilon )= \frac{\epsilon (1-r_{1})}{r_{1}}, $$

then, by the boundedness of $\mathcal{P}_{1}$, we conclude that

$$ \begin{aligned} & \flat \bigl(\mathfrak{G}_{1} \mathcal{P}_{1}(\chi _{1}, \chi _{1},\chi _{i})\bigr)\\ &\quad = \frac{\mathfrak{G}_{1}\mathcal{P}_{1}(\chi _{1},\chi _{1},\chi _{i})}{r_{1}} \leq \mathfrak{G}_{1}(\chi _{1},\chi _{2},\chi _{3}) \\ &\quad\leq \mathfrak{G}_{1}(\chi _{1},\chi _{1},\chi _{i})+\mathfrak{G}_{1}( \chi _{2},\chi _{2},\chi _{j})+\mathfrak{G}_{1}(\chi _{3},\chi _{3}, \chi _{k}) \\ &\quad= \flat \bigl(\mathfrak{G}_{1}(\chi _{1},\chi _{1},\chi _{i})\bigr)-\wp \bigl( \mathfrak{G}_{1}( \chi _{1},\chi _{1},\chi _{i})\bigr)+ \mathfrak{G}_{1}( \chi _{2},\chi _{2},\chi _{j})+\mathfrak{G}_{1}(\chi _{3},\chi _{3}, \chi _{k}) \\ &\quad\leq \flat \bigl(\mathfrak{G}_{1}(\chi _{1},\chi _{1},\chi _{i})\bigr)-\wp \bigl( \mathfrak{G}_{1}( \chi _{1},\chi _{1},\chi _{i})\bigr) \\ &\qquad{} +\min \bigl\{ \mathfrak{G}_{1}(\chi _{2},\chi _{2},\mathcal{P}_{1} \chi _{2}), \mathfrak{G}_{1}(\chi _{2},\chi _{2}, \mathcal{P}_{1}\chi _{1}), \mathfrak{G}_{1}(\chi _{1},\chi _{1},\mathcal{P}_{1}\chi _{1}), \mathfrak{G}_{1}(\chi _{1},\chi _{1}, \mathcal{P}_{1}\chi _{2}) \bigr\} . \end{aligned} $$

Hence, one obtains the inequality (12). Similarly for $\mathcal{P}_{2}$, which shows that in view of Lemma 3.1, the integral operator $\mathcal{P}$ has a unique fixed point lying in $B_{r}=(B_{r1},B_{r2})$, $r\leq 1$. □

### Control solution

Here, we investigate the controlling of the unique solution under the assumptions of Theorem 3.3. We construct the modest control system based on system (11). For a linear case $X(t,\chi (t))=\chi (t)$ and $A(t,\Lambda (t))= \Lambda (t)$, system (11) becomes

$$ \begin{aligned} & {\mathcal{D}}^{\nu }\chi (t) =\sigma _{1}(\nu,t) \chi (t)+ \sigma _{0} (\nu,t) \chi (t)+ \sigma (t) \Lambda (t), \\ & {\mathcal{D}}^{\mu }\Lambda (t) =\rho _{1}(\mu,t) \Lambda (t)+\rho _{0} (\mu,t) \Lambda (t)+ \rho (t) \chi (t). \end{aligned} $$

By considering the formula of $\sigma _{1}$ and $\sigma _{0}$ and of $\rho _{1}$ and $\rho _{0}$, we have

15 $$\begin{aligned} \begin{aligned} &{\mathcal{D}}^{\nu }\chi (t) = \bigl[ (1-\nu ) t^{\nu }+ \nu t^{1- \nu }\bigr]\chi (t)+ \sigma (t) \Lambda (t), \\ &{\mathcal{D}}^{\mu }\Lambda (t) = \bigl[ (1-\mu ) t^{\mu }+ \mu t^{1-\nu }\bigr] \Lambda (t)+ \rho (t) \chi (t), \end{aligned} \\ \begin{aligned} &{\mathcal{D}}^{\nu }\chi (t) = \biggl( \frac{t^{-\nu }}{1+t^{-\nu }} \biggr) \chi _{{\nu }}(t)+\sigma (t) \Lambda (t), \\ &{\mathcal{D}}^{\mu }\Lambda (t)= \biggl( \frac{t^{-\mu }}{1+t^{-\mu }} \biggr) \Lambda _{{\mu }}(t) +\rho (t) \chi (t), \end{aligned} \end{aligned}$$

where

$$ \chi _{\nu }(t)= ((1-\nu )t^{2\nu } \bigl(1+t^{-\nu } \bigr)+ (\nu t) \bigl(1+t^{- \nu } \bigr) $$

and

$$ \Lambda _{\mu }(t)= ((1-\mu )t^{2\mu } \bigl(1+t^{-\mu } \bigr)+ (\mu t) \bigl(1+t^{- \mu } \bigr). $$

This control models a diffusion of cells, which we aim to minimize. To complete the minimization, we define the following norm:

$$ \bigl\| H (\Upsilon _{1},\Upsilon _{2})\bigr\| _{\infty }=\sup _{t} \bigl( \bar{\sigma }\bigl(\Upsilon _{1}(t) \bigr), \bar{\rho }\bigl(\Upsilon _{2}(t)\bigr) \bigr), $$

where *σ̄* represents the maximum singular value of $\Upsilon _{1}$ and *ρ̄* indicates the maximum singular value of $\Upsilon _{2}$. The problem of the *H*-controller is to select $(\chi _{\nu },\Lambda _{\mu })$ that makes the closed-loop system internally stable, i.e. minimize the value $\|H\|_{\infty }$. In our discussion, we let $\Upsilon _{1}(t)=\chi (t)$ and $\Upsilon _{2}(t)=\Lambda (t)$. Also, we consider that there exist two Perron functions $\omega _{1}(\cdot,\cdot), \omega _{2}(\cdot,\cdot)$ such that

$$\begin{aligned} &\mathrm{(A3)}\quad \sup_{t} \bigl( \bar{\sigma }\bigl(\Upsilon _{1}(t)\bigr), \bar{\rho }\bigl( \Upsilon _{2}(t)\bigr) \bigr)\\ &\phantom{{(A3)}\quad}\quad \leq \bigl( \omega _{1}\bigl(t,|\chi _{\nu,1}- \chi _{\nu,2}|\bigr), \omega _{2}\bigl(t,|\Lambda _{\mu,1}-\Lambda _{\mu,2}|\bigr) \bigr)+\varepsilon,\quad \varepsilon \in [0,1]. \end{aligned}$$

Recall, a function *ω* is called a Perron function if it is integrally bounded on bounded sets, $\omega (t, 0) = 0, \omega (t,.) $ is non-decreasing for every *t* and the zero function is the only solution of the scalar differential equation $f'(t) = \omega (t, f(t)), f(0) = 0$. System (15) satisfies the following functional system:

16 $$ \begin{aligned} & \chi (\cdot) \rightarrow \int _{0}^{T} \biggl( \biggl( \frac{t^{-\nu }}{1+t^{-\nu }} \biggr) \chi _{{\nu }}(t)+\sigma (t) \Lambda (t) \biggr) \,d_{\nu }t, \\ &\Lambda (\cdot)\rightarrow \int _{0}^{T} \biggl( \biggl( \frac{t^{-\mu }}{1+t^{-\mu }} \biggr) \Lambda _{{\mu }}(t) +\rho (t) \chi (t) \biggr) \,d_{\mu }t, \end{aligned} $$

which is continuous in $B_{r} $. Moreover, the above integral function is the lower semi-continuous version of the function

$$\begin{aligned} ( \int \bigl( \sigma _{1}(\nu,t) \chi (t)+\sigma _{0} ( \nu,t) X\bigl(t, \chi (t)\bigr)+\sigma (t) \Lambda (t) \bigr) \,d_{\nu }t+c_{1}, \\ \int \bigl( \rho _{1}(\mu,t) \Lambda (t)+\rho _{0} ( \mu,t) A\bigl(t, \Lambda (t)+ \rho (t) \chi (t) \bigr) \,d_{\nu }t+c_{2} \bigr). \end{aligned}$$

But the limit solution set is $B_{r} $, which is compact and every lower semi-continuous real valued function attains its minimum to a compact set. Thus, we seek the following optimal control theorem.

#### Theorem 3.4

*Let the assumptions* (A1)–(A3) *hold*. *Then system* (11) *admits an optimal limit solution*.

#### Remark 3.5

**Figure 2:**
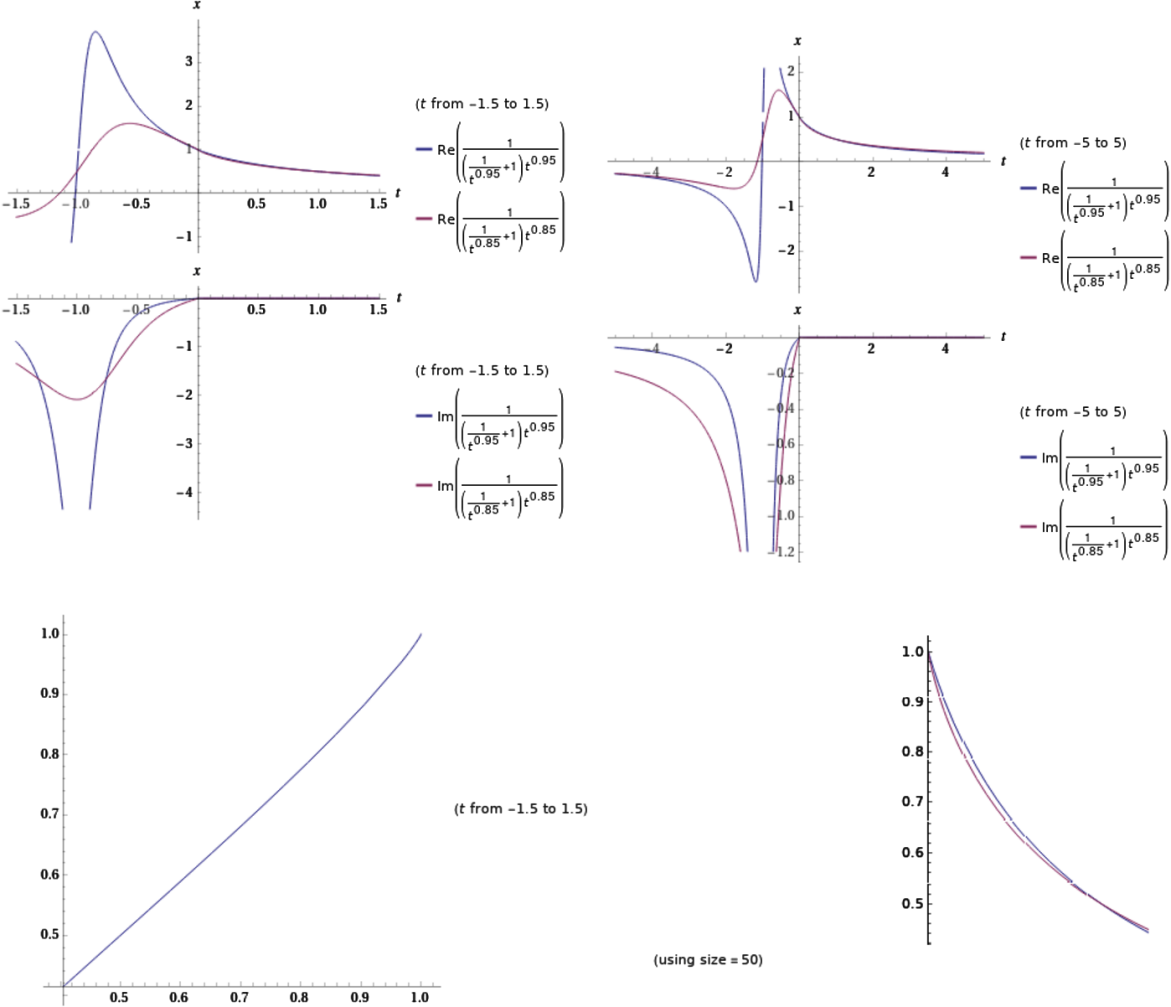
The optimal values of the controller at the fractional parameters $\nu =0.85$ and $\mu =0.95$

**Figure 3:**
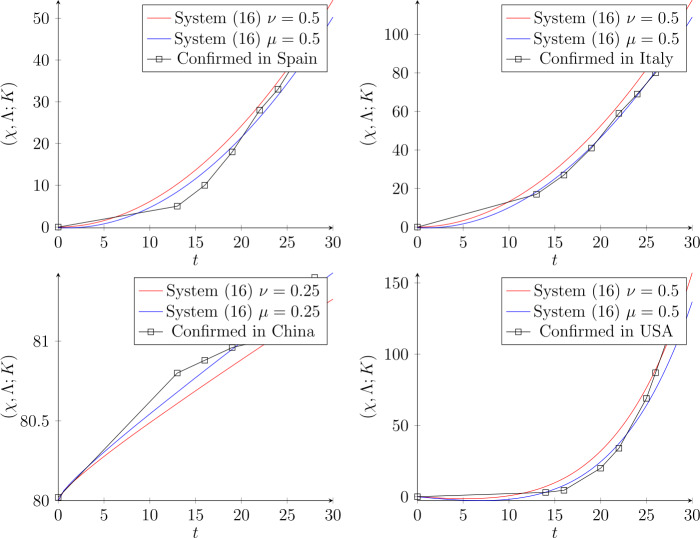
The dynamic process of system (16) with the connections $\sigma (t)=0.012 t$ and $\rho (t)=0.12(t+1)$ for Spain data. Meanwhile for Italy data, which is rapidly increasing, we use $\sigma (t)=0.026 t$ and $\rho (t)=0.26(t+1)$. China data in March have a stable distribution, therefore we suggest $\nu =\mu =0.25$ with $\sigma (t)=80+0.033 t$ and $\rho (t)=0.34 t+80 $. Finally, for the USA, the graph shows high conformation, thus we utilize exponential connections

- The controller protects the set-point as the disturbance $(\sigma,\rho )$ is changed. The system with integral control jumps to oscillation as there is reduction of $(\sigma,\rho )$. The presentation of a controller appears to make the solutions of the coupled system less stable, or more oscillatory, than for the system without supplementary control.
- The model described the relation between the symptomatic growing detected people $\chi (t) $ and the asymptomatic one, $\Lambda (t) $, we established a unique relation under the solution of Theorem 3.3. Furthermore, one catches beneficial signs on the number of asymptomatic persons.
- The dynamical method may be such that the macroscopic explanation indicates the possibility of assessing the accumulative number of asymptomatic persons as a function of time. We recall that our study applies to the temporary region of fast growth and not to the satiety phase of the diffusion. As exposed in Fig. 3, in the short-lived rule, the increasing number of asymptomatic people turns out to be larger than the number of infected people, but smaller than in other planned simulations [3].
- Biological importance: agreeing with the standard control, the system possesses the focuses of positive complexes within acceptable limits and thus gives the internal stability of growing cells and creatures (see [11]). Our conclusions clarify how the integral control allows biological systems to continue full-bodied under the ordinary circumstances, even when they demonstrate periodic or chaotic performance. In other words, it illustrates that an internal parameter in contradiction of external disturbances (parameter changes) can be conserved even when systems are wavering. Integral control leads to a full life controlling device that covers more than the stability of the unique solution.
- The optimal value of the fractional parameters indicates that $t^{-\nu }/(1+t^{-\nu }) \rightarrow 1$ and $t^{-\mu }/(1+t^{-\mu }) \rightarrow 1$, when $\nu =0.85$ and $\mu =0.95$. Figure 2 shows the plot of these controller factors.

## Applications

In this section, we start to exam our system by suggesting real data from the internet. Figure 3 indicates the conformed data in March for the highest-value countries. We used Mathematica Wolfram 11.2 for calculation and coding the system.

Generally, in the diffusion of infections there are a huge number of asymptomatic residents, which affects the time dependence of $\chi (t)$ in the temporary phase. Both $\chi (t)$ and $\Lambda (t)$ approach a steady-state condition after some time, challenging as regards assessing by mathematical reproductions, where asymptomatic persons are still current, but with a low pathological load. From this point of view, the asymptomatic people should show degeneration in the fullness phase. The optimal control shows its convergence of the actual data, under different sets of parameters.

Our set of parameters plays an important role to detect the suitable graph of data. As is seen, different connections (coefficients) are suggested to discover and cover the real data. For instance, USA data require an exponential connection of $\sigma (t)$ and $\rho (t)$, while China connections are linearly selected. The dynamic process of system (16) with the connections $\sigma (t)=0.012 t$ and $\rho (t)=0.12(t+1)$ are for Spain data. Meanwhile for Italy data, which is rapidly increasing, we use $\sigma (t)=0.026 t$ and $\rho (t)=0.26(t+1)$. Moreover, the guaranteeing set of parameters implies the suitable graph of the actual data for both $\chi _{\nu }(t)$ and $\Lambda _{\mu }(t)$. Finally, the suggested dynamic system and its integral control system provide an opportunity of deep study and flexibility for some modifications and extensions depending on the set of data (see Theorem 3.4). For example, if one aims to study the oscillation of solutions, it is better to use connections formula based on the $\cos (t)$ and $\sin (t)$ functions (Fig. 3).

## Conclusion

From above, we confirm that the use of CC has the flexibility to control the orbit of the solution. The CC operation involves the controller term with various types of connections functions (fractional connections). Some of these connections are expressed in terms of linear functions and others are nonlinear functions. The existence and uniqueness of the dynamical system of the growth are established by using a self-function. The solvability is studied by using a fixed point theorem of a metric space. The control solution is described by using an integration formula. One can suggest it as a maximum solution.

## Data Availability

Not applicable.
